# Perspectives from the Society for Pediatric Research: Probiotic use in urinary tract infections, atopic dermatitis, and antibiotic-associated diarrhea: an overview

**DOI:** 10.1038/s41390-020-01298-1

**Published:** 2020-12-07

**Authors:** Catherine S. Forster, Michael H. Hsieh, Michael D. Cabana

**Affiliations:** 1 Department of Pediatrics, Children’s National Health System, Washington, DC USA; 2 Department of Pediatrics, Children’s Hospital at Montefiore and the Albert Einstein School of Medicine, Bronx, NY USA

## Abstract

Probiotics have received significant attention within both the scientific and lay communities for their potential health-promoting properties, including the treatment or prevention of various conditions in children. In this article, we review the published data on use of specific probiotic strains for three common pediatric conditions: the prevention of urinary tract infections and antibiotic-associated diarrhea and the treatment of atopic dermatitis. Research into the utility of specific probiotic strains is of varying quality, and data are often derived from small studies and case series. We discuss the scientific merit of these studies, their overall findings regarding the utility of probiotics for these indications, issues in reporting of methods, and results from these clinical trials, as well as future areas of investigation.

## Impact

- Review of data for commonly encountered diagnoses in the general pediatric population.
- Highlights gaps in the evidence and provides insight into new avenues of research.

## Introduction

A growing understanding of the microbiome has led to a broader understanding of both health and disease. The advent of affordable advances in technology has made the ability to identify bacteria on the basis of 16S rRNA sequencing feasible, leading to the discovery of the populations of bacteria both on and within the body.^1–3^ This prompted the Human Microbiome Project, the goal of which was to study the role of these various microbial communities.^4^ Much of the early work of the Human Microbiome Project focused on documenting the components of site-specific microbiomes during a normal, healthy state. More recently, investigators have begun to explore correlations between perturbations in the microbiome (e.g., dysbiosis) with specific diseases and determine the therapeutic potential of administration of exogenous bacteria or yeast (e.g., probiotics) in various conditions.

In an effort to standardize the definition of probiotics, a 2014 consensus panel recommended the following amended definition: “Probiotics are live organisms that, when given in adequate amounts, confer a health benefit to the host.”^5^ While this concept has gained recent popularity, it was first described in the early twentieth century by Elie Metchnikoff, who hypothesized that live bacterial cultures found within yogurt had health-promoting benefits.^6^ Although quiescent for more than half a century, the notion of a therapeutic role of probiotics has gained relatively recent traction, with the probiotics market estimated to have exceeded $35 billion in 2015.^7^ The field of probiotics research is still growing and is rife with studies that include small numbers of patients, use different probiotic strains, and have mixed results regarding the utility of probiotics. Further, the concept of strain specificity, in which effects of the probiotic in question are attributable to a specific strain that are not necessarily present in other strains of the same bacteria or yeast, has caused further complexity in this area of research given the lack of strain data present in early work on probiotics.^8^

The purpose of this review is to examine the literature on the use of probiotics in children for three common, general pediatric conditions (prevention of urinary tract infections (UTI), treatment of atopic dermatitis (AD), and prevention of antibiotic-associated diarrhea (AAD)) and to highlight future areas of research.

## Methods

We conducted a scoping review to identify peer-reviewed publications of randomized controlled trials (RCTs) focused on the prevention of UTIs and AAD and the treatment of AD. We searched PubMed and Scopus from January 1, 1994 to August 1, 2020. The search included the terms “probiotics,” “diarrhea,” “atopic dermatitis,” “urinary tract infections,” and “child.” All terms were searched as both keywords and Medical Subject Heading (MeSH) terms in PubMed (National Library of Medicine; Bethesda, MD) and as keywords in Scopus (Elsevier; Philadelphia, PA).

We included peer-reviewed publications of RCTs that focused on a pediatric population >3 years of age. If the range of included children overlapped with 3 years, we used the mean age of patients to determine inclusion such that trials where the mean age of the population was >3 years were included. This age was specifically chosen to exclude studies that focused exclusively on neonates, infants, or pregnant women. We excluded studies if the primary outcome was neither incidence or severity of AAD, clinical improvement in AD, or incidence of UTI; if the outcome was not reported in terms of clinical change; if the intervention was a probiotic product without a clearly specified probiotic organism (e.g., yogurt); if the intervention was a synbiotic or prebiotic; or if the outcome was not relevant to the general pediatrics population. For AAD studies, we excluded those that focused exclusively on an outcome of *Clostridium difficile* colitis. Studies that used yogurt as an intervention but specified both the probiotic strains and amounts of each probiotic within the yogurt were included.

## Results

**Fig. 1:**
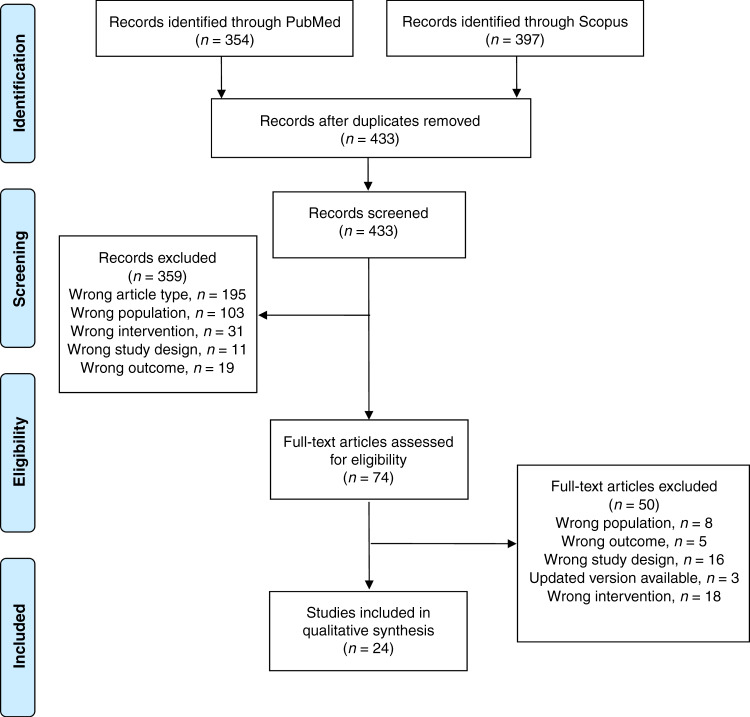
PRISMA diagram of articles within this review. This figures depicts the number of articles initially identified, those exluded and their reasons, and the number of articles ulimately included in this scoping review.

The search resulted in 354 articles in PubMed and an additional 78 citations (after exclusion of duplicates) from Scopus. Three hundred and sixty six of these articles were excluded, leaving 74 for full-text review. We reviewed those 74 articles, 50 of which were excluded at the full-text review stage. The remainder were those that addressed the population, intervention, and outcomes of interest in this review (Fig. 1).

### Urinary tract infections

There are limited pediatric studies focused on UTI prevention in children. We found only two RCTs on this topic. Each study used a different probiotic intervention. In both studies, the children were at risk for UTI based on vesicoureteral reflux or a history of UTI.

One study examined the utility of a combination of *Lactobacillus acidophilus (*LA5) and *Bifidobacterium lactis* (BB12) and antibiotics versus antibiotics alone. In this trial, 106 children with primary persistent vesicoureteral reflux were randomized to either prophylaxis with probiotics and nitrofurantoin or nitrofurantoin alone. There was no significant difference in the incidence of recurrent UTI between groups, but there was a lower incidence of febrile UTIs in year 3 in the probiotics group. This study, however, was limited by use of a positive urine culture as the definition of UTI, which may bias the results given that a positive urine culture does not always reflect a UTI.^9^ More recently, a randomized double-blind placebo-controlled trial of 244 children (181 who completed the trial) with a single, uncomplicated febrile UTI were randomized to receive either a combination of multiple strains of *Lactobacillus* (including *L. acidophilus* and *Lactobacillus rhamnosus*, strains not specified)) and *Bifidobacterium* species (including *Bifidobacterium bifidum* and *B. lactis*, strains not specified) or placebo following treatment of the acute UTI. Children in the probiotic group had a significantly lower rate of UTI recurrence after 18 months compared to children in the placebo group.^10^

While one study did provide some evidence that a probiotic intervention may have utility for UTI prophylaxis, further work is necessary to confirm these results (Table 1).

**Table 1:** Summary of articles examining the effects of probiotics in children with urinary tract infections.

Author (year) and study design	Setting	Population	Probiotic used	Dose	Duration		Outcome
Mohseni et al.^9^ (2013) RCT: probiotic yogurt and antibiotic versus antibiotic alone	Outpatient clinic (Children’s Medical Center, Tehran University of Medical Sciences) Tehran, Iran	106 children (3–15 years) with unilateral VUR and history of recurrent UTIs	*Lactobacillus acidophilus* (LA5)	10^7^ CFU TID	Months 1–6	2.5 months of probiotics followed by 15 days off probiotics	No difference in incidence (per person per year) of UTIs between groups for years 1–3 (incidence in the probiotics and antibiotics groups, 1.29, 0.70, 0.51; incidence in antibiotics alone 1.54, 1.13, 0.95). Fewer febrile UTIs in year 3 in the probiotics and antibiotics group (0) versus antibiotics alone (0.13)
			*Bifidobacterium lactis* (BB12)		Months 7–12	45 days on probiotics followed by 15 days off	
					Months 13–18	1 month on probiotics followed by 1 month off	
					Months 19–24	15 days on probiotics followed by 45 days off of probiotics	
Sadeghi-bojd et al.^10^ (2019) RCT: combination of probiotics versus placebo	Four outpatient clinics (Kurdistan University of Medical Sciences, Mashhad University of Medical Sciences, Semnam University of Medical Sciences, and Zahedan University of Medical Sciences), Iran	181 children (4 months–5 years) after first acute non-complicated febrile UTI	Combination of 11 different strains of both *Lactobacillus* and *Bifidobacterium*. Strains not specified	500 mg capsule, each strain with different dose	18 months		Difference in rate of UTIs between groups. 3/91 children (3%) in the probiotic group and 15/90 children (17%) in the placebo group had recurrent UTI

*UTI* urinary tract infection, *CFU* colony-forming unit, *BID* twice daily, *VUR* vesicoureteroreflux.

### Atopic dermatitis

We found ten studies focused on probiotic interventions for AD treatment in children. It was not possible to combine the results of the studies, as each of the studies used a different probiotic intervention or treated children with varying degrees of AD severity, based on the reported SCOring Atopic Dermatitis (SCORAD) severity scores, previous medications used, or other descriptors.

There are several trials that focus on the effectiveness of single probiotic strains for AD. Han et al. randomized 118 children (83 who completed the study) to 12 weeks of *Lactobacillus plantarum* CJLP133 or placebo. Those in the probiotic group had a significant decrease in the SCORAD index^11^ at 14 weeks compared to the control group. However, this difference was no longer present at 16 weeks, suggesting that any potential beneficial effect of the probiotic does not persist after the discontinuation of therapy.^12^

Prakoeswa et al. randomized 22 patients <14 years of age with mild-to-moderate AD and elevated levels of total serum immunoglobulin E (IgE) to receive 12 weeks of either *L. plantarum* IS-10506 or placebo. They reported decreases in SCORAD in both groups, although a larger decrease in those receiving probiotics than placebo.^13^

In another randomized double-blind placebo controlled study (*n* = 88 enrolled and 75 who completed the study), Woo et al. assessed the effectiveness of *Lactobacillus sakei* KCTC10755BP for 12 weeks and found that children 2–10 years of age in the treatment group had improved SCORAD at 12 weeks compared to placebo.^14^ However, a potential limitation of this work is that patients in the probiotic group had lower baseline levels of both cytokines C-C chemokine motif ligand 17 (CCL17) and CCL27, both of which are correlated with SCORAD.^14^

There have also been several studies focusing on various combinations of species. Wang et al. conducted a double-blind, randomized-controlled trial comparing the effectiveness of *Lactobacillus paracasei* GMNL-133, *Lactobacillus fermentum* GM090, or a combination of both, compared to placebo for 3 months for 220 children (212 who completed the study) aged 1–18 years with AD. They found significantly reduced disease severity, based on SCORAD, in all three probiotic groups compared to placebo.^15^ Sistek et al. randomized 59 children (49 who completed all 16 weeks of the study) aged (1–10 years) with SCORAD ≥10 and either a positive skin prick test or positive radioallergosorbent test to a common food or environmental allergen to receive either 12 weeks of a combination of probiotics (*L. rhamnosus* and *B. lactis*, strains not specified) or placebo. After adjusting for baseline differences in SCORAD between groups, the authors found that only food-sensitized children in the probiotic group had a significant improvement in their SCORAD.^16^

Navarro-Lopez et al. conducted a double-blind placebo-controlled study and randomized 50 children (47 of whom were analyzed) 4–17 years of age to receive either a combination of *B. lactis* CECT 8145, *Bifidobacterium longum* CECT 7347, and *Lactobacillus casei* CECT 9104 or placebo for a period of 12 weeks. Those assigned to the probiotics arm had significant improvements in disease severity and decreased use of topical steroids compared to the placebo arm, although no changes were seen in any serum markers.^17^ However, of note, all children in the study were required to consume a high-quality Mediterranean Diet, as defined by a Mediterranean Diet Quality Index score >7,^18^ which may limit the generalizability of this study.^17^

Yesilova et al. conducted a double-blind, randomized placebo-controlled study with children (*n* = 40, 39 of whom completed the study) and compared the effectiveness of a combination of *Bifidiobacterium bifidium*, *L. acidophilus*, *L. casei*, and *Lactobacillus salivarius* (strains not specified) for a period of 8 weeks. While the combination of probiotics significantly reduced disease severity, the baseline SCORAD differences between the placebo and probiotic groups were much greater than the post-intervention SCORAD, which may be due to a regression to the mean phenomenon, rather than a true difference in effectiveness.^19^

Three other studies did not find a benefit to probiotic use. Yang et al. conducted a randomized study of children with mild-to-moderate AD (*n* = 100 enrolled, 71 completed the study). They found that the combination of *L. casei*, *L. rhamnosus*, *L. plantarum*, and *B. lactis* (strains not specified) did not demonstrate an improvement in the probiotic group compared to the placebo.^20^

Rosenfeldt et al. used a crossover, placebo-controlled design and compared lyophilized *L. rhamnosus* 19070-2 and *L. reuteri* DSM 122460 to placebo. They found that patients with an allergic phenotype (defined as one positive skin prick response and elevated IgE levels) did have a significant reduction in SCORAD score during probiotic, but not placebo, treatment. Of note, the washout period was 6 weeks, which is likely sufficient in this context, although contamination and insufficient washout may confound the results.^21^ More recently, investigators randomized 95 children (82 who completed the study) to either *L. pentosus* (strain not specified) or placebo. While significant improvement was seen in SCORAD in both groups, there was no difference in the degree of improvement between the intervention and placebo groups^22^ (Table 2).

**Table 2:** Summary of articles examining the effects of probiotics in children with atopic dermatitis.

Author (year) and study design	Setting	Population	Probiotic used	Dose	Duration	Outcome
Han et al.^12^ (2012) RCT: probiotic versus placebo	Chung-Ang University Hospital (specific site of recruitment not specified) Seoul, Korea	83 patients (1–13 years of age) with SCORAD 20–50	*L. plantarum* CJLP133	0.5 × 10^10^ CFU BID	12 weeks	Significant improvement in SCORAD at 14 weeks, but improvement did not persist at 16 weeks. Mean (SD) 14-week SCORAD: 20.4 (11.8) in the probiotics group versus 25.6 (11.6) in the placebo group (*p* = 0.044). 16-week SCORAD: 21.9 (13.2) for the probiotics group and 24.9 (10.7) for the placebo group (*p* = 0.261)
Prakoeswa et al.^13^ (2017) RCT: probiotic or placebo	Outpatient clinic, Allergy Immunology Division of the Dermatology and Venereology Department, Universitas Airlangga/Dr. Soetomo, Surabaya, Indonesia	22 patients (0–14 years of age) with mild-to-moderate AD and elevated levels of total serum IgE	*L. plantarum* IS-10506	10^10^ CFU/day	12 weeks	All groups had a decrease in SCORAD, but the decrease was greater in the probiotic group. Mean (SD) SCORAD in the probiotic group changed from 55.3 (15.3) to 18.53 (14.20), compared to 48.9 (13.8) to 22.04 (8.82) for the placebo group
Woo et al.^14^ (2010) RCT: probiotic versus placebo	Department of Pediatrics, Chungbuk National University Hospital, Cheongju, Korea	75 children (2–10 years of age) with SCORAD ≥25 whose SCORAD did not change ≥10% within 2 weeks of inclusion	*L. sakei* KCTC10755BP	5 × 10^9^ CFU BID	12 weeks	Decrease in SCORAD seen in both groups from baseline. SCORAD levels in the probiotic group lower at week 12 than placebo. Mean pretreatment adjusted SCORAD at 12 weeks was 28.8 for the probiotic groups and 35.8 in the placebo group
Wang and Wang^15^ (2015) RCT: one of the three different probiotics or placebo	Pediatric outpatient clinics of Taipei Hospital Ministry of Health and Welfare, Taiwan	212 children 1–18 years of age with SCORAD >15 and positive skin prick or elevated IgE to common allergens	*L. paracasei* GMNL-133	2 × 10^9^ CFU/day	12 weeks	All groups had a decrease in SCORAD at 12 weeks. Children in all 3 probiotic groups had lower mean SCORAD at week 12 compared to those in the placebo group. Mean (SD) 12-week SCORAD was: 25.62 (22.35) for *L. paracasei*, 28.38 (20.43) for *L. fermentum*, 24.17 (17.63) for the combo, and 39.39 (18.34) for the placebo group
			*L. fementum* GM090			
			Combination of *L. paracasei* GMNL-133 and *L. fermentum* GM090	4 × 10^9^ CFU/day		
Sistek et al.^16^ (2006) RCT: probiotic versus placebo	Greater Wellington area, New Zealand	59 children (1–10 years) with SCORAD ≥10 and either a positive skin prick test or positive RAST to a common food or environmental allergen	Combination of *L. rhamnosus* and *B.* *lactis* Strains not specified	2 × 10^10^ CFU/day	12 weeks	All groups had decrease in SCORAD, but significant improvement only seen in the in food-sensitized children in the probiotic group. Geometric mean ratios (95% CI) of SCORAD values at 16 weeks in food-sensitized group: 0.76 (0.59–0.97) for the probiotic group, 0.83 (0.66–1.05) for placebo; non-food-sensitized: 0.83 (0.55–1.27) for probiotic, 0.89 (0.53–1.52) for placebo
Navarro-Lopez et al.^17^ (2018) RCT: probiotic and standard care versus placebo and standard care	Outpatient pediatric dermatology clinic, Centro Dermatologico Estetico de Alicante, Alicante, Spain	47 children between 4 and 17 years of age with SCORAD between 20 and 40 who were using topical steroids and consuming a Mediterranean diet	Combination of: *Bifidobacterium lactis* CECT 8145, *B longum* CECT 7347, and *L casei* CECT 9104	10^9^ CFU/day	12 weeks	Greater decrease in SCORAD in the probiotic group compared to the placebo group, and reduction of topical steroid use to treat flares in the probiotic group compared to the placebo group. The change in SCORAD from baseline at week 12: −27.0, 95% CI (−31.1, −22.8) for the probiotic group −7.8, 95% CI (−11.9, −3.6) for placebo
Yesilova et al.^19^ (2012) RCT: combination of probiotics or placebo	Turkey (specific location not specified)	39 patients (1–13 years of age) with moderate-to-severe AD	*Bifidiobacterium bifidium*, *L. acidophilus*, *L. casei*, and *L. salivarius* Strains not specified	2 × 10^9^ CFU /day	8 weeks	All groups had a decrease in SCORAD, but the decrease was greater in the probiotic group. Mean (SD) post-SCORAD in the probiotic group decreased from 35.4 (13.4) to 12.4 (7.2); in the placebo group decreased from 28.1 (6.1) to 15.3 (5.1)
Yang et al.^20^ (2014) RCT: combination of probiotic or placebo	Pediatric Allergy and Respiratory Center of Soonchunhyang University Hospital, Seoul, Korea	71 children (2–9 years of age) with SCORAD ≤40	Combination of: *L. casei*, *L. rhamnosus, L. plantarum*, and *B. lactis* Strains not specified	1 × 10^9^ CFU of each strain BID	6 weeks	Significant improvement in both groups, but no difference in the degree of improvement between the probiotic and placebo groups. EASI in the probiotic group decreased from median (IQR) of 7.2 (4.8–10.3) to 4.7 (2.2–6.8) at 6 weeks: placebo: 8.3 (4.7–10.6) to 4.5 (2.6–9.0) at 6 weeks
Rosenfeldt et al.^21^ (2003) Randomized cross-over trial: probiotic versus placebo	Outpatient pediatric clinic at Hvidovre Hospital and the Department of Dermatology Clinic at Gentofte Hospital, Denmark	43 patients (1–13 years of age) with AD who had not been treated with systemic steroids	Combination of *L. reuteri* DSM 122460 and lyophilized *L. rhamnosus* 19070–2	10^10^ CFU of each strain BID	6 weeks	No significant improvement in SCORAD in either group. Patients with an allergic phenotype (positive skin prick response and elevated IgE levels) did have a significant improvement in SCORAD. Mean (95% CI) change in SCORAD after probiotic exposure: −3.4 (−7.9, 1.1); after placebo exposure: 0.5 (−3.8, 3.8). Change in subset of allergic patients: −2.4 (−7.5, 2.8) after probiotic exposure; 3.2 (−1.6, 7.9) after placebo
Ahn et al.^22^ (2020) RCT: probiotic versus placebo	Department of Pediatrics, Korea University Anam Hospital, Seoul, Korea	81 patients (2–13 years of age) with mild-to-moderate AD	*L. pentosus*	1 × 10^10^ CFU BID	12 weeks	Significant improvement in both groups, but no difference in degree of improvement between the probiotic and placebo groups. Mean (SD) SCORAD in the probiotic group decreased from 30.4 (8.6) to 23.6 (11); in the placebo group decreased from 34.3 (8.3) to 23.1 (9.7)

*CFU* colony-forming unit, *BID* twice daily *SD* standard deviation, *95% CI* 95% confidence interval, *SCORAD* SCORing Atopic Dermatitis, a scale used to determine severity of clinical symptoms.

A recent meta-analysis reported the positive effect of probiotics on decreasing disease severity in children with AD.^23^ However, a recent Cochrane review noted a difference in effects based on age: while children between 2 and 12 years of age had significant improvement in SCORAD with use of probiotics, this was not seen in children under the age of 2 years.^24^

### Antibiotic-associated diarrhea

We found 13 studies focused on probiotic interventions for the prevention of AAD. The most common probiotic interventions tested included *Saccharomyces boulardii* (4 studies), *Lactobacillus reuteri* DSM 17938 (2 studies), and *Lactobacillus GG* (2 studies). All the studies include children who were prescribed antibiotics in an outpatient setting and usually within 48 h.

The majority of studies focused on single strains of probiotics, the most studied of which is *S. boulardii*. A study by Kotowska et al. randomized 269 children (246 of whom were analyzed) who were prescribed antibiotics for either acute otitis media or respiratory infections to either probiotic or placebo. They found a lower rates of AAD in the probiotics versus placebo group (3.4 versus 17.3%).^25^ Similarly, Jindal et al. randomized 600 children given antibiotics to receive either probiotics and antibiotics or antibiotics alone. They also report a lower rate of AAD in the probiotics group compared to antibiotics alone (5.3 versus 24.0%). Of note, this study did not include a placebo group.^26^ A 2013 study by Shan et al. found that patients who received antibiotics for lower respiratory tract infections and also received *S. boulardii* (*n* = 167) had a lower incidence of AAD than those who were not given probiotics (*n* = 166), with a relative risk of 0.22 (95% confidence interval (CI): 0.1–0.5). Further, this group also reported that initiation of *S. boulardii* in children who developed AAD but were not initially randomized to receive probiotics resulted in a decreased stool frequency compared to those in whom probiotics were not initiated. However, the authors in this work included diarrhea due to *C. difficile* in their AAD group, potentially affecting the interpretation of their results.^27^ A final study by Bin et al. found a lower incidence of AAD in children receiving triple antibiotic therapy for *Helicobacter pylori* infection in children who were given *S. boulardii* (CNCM I-745) with the antibiotics compared to patients who received antibiotics alone (11.8 versus 28.3%, *p* < 0.05).^28^

Other authors have focused on single strains of *Lactobacillus*, with mixed results. A recent multi-center study of 438 children (447 children enrolled) randomized children to either *L. plantarum* DSM9843 (LP299V)) (*n* = 218) or placebo (*n* = 220) and found that the use of probiotics did not reduce the incidence of AAD or the mean number of loose, watery stools in children given antibiotics by their primary care pediatrician.^29^ Two studies focused on *L. reuteri* DSM 17938, neither of which found a difference in the incidence of AAD.^30,31^ However, in one of these studies, the overall rate of AAD in the entire cohort was quite low at 2%, which likely contributed to their negative findings.^30^ Similar work has shown that *Lactobacillus casei* sps. *rhamnosus* (LGG) is beneficial, when compared to placebo, for reduction of stool frequency in children on oral antibiotics.^32^ A final, older study, focused on *Lactobacillus casei* sps. *rhamnosus* (*Lactobacillus GG*) and found that it decreased the incidence of AAD compared to placebo.^33^

Other studies have used combinations of strains. One study of a combination of strains of *L. rhamnosus* (strains E/N, Oxy, and Pen) found that children on antibiotics for common illnesses in the probiotic (*n* = 120), as opposed to placebo (*n* = 120), group had a significant reduction of all diarrhea, defined as three or more episodes of loose and watery stools per day for ≥48 h that occurred during or up to 2 weeks following antibiotic therapy. However, there was no difference in the incidence of AAD (including diarrhea due to *C. difficile*) between the two groups.^34^ Another double-blind, placebo-controlled trial studied the combination of *B. longum* PL03, *L. rhamnosus* KL53A, and *L. plantarum* PL02 in 78 children exposed to antibiotics and did not find a difference in the incidence of AAD. They did note, however, a significant reduction in the number of episodes of diarrhea in the probiotic group compared to placebo.^35^ Another study used a different combination of probiotics that included *Lactobacillus sporogenes*, *Streptococcus faecalis* T110, *Clostridium butyricum* TO-A, and *Bacillus mesentericus* TO-A. In this work, in which the probiotic mixture was given twice a day to patients on amoxicillin–clavulanate for an unspecified amount of time, there was a decreased incidence in AAD compared to children who received antibiotics without the probiotic mixture.^36^ Others compared a probiotic yogurt containing *Lactobacillus GG* ((strain deposit number (SDN): ATCC53103), *B. lactis* (SDN: DSM15954), and *L. acidophilus* (SDN: DSM13241) with a pasteurized yogurt containing *Streptococcus thermopolis* and *Lactobacillus bulgaricus*. In this study, the authors found a decrease in both the incidence of AAD and incidence of severe diarrhea in the probiotic groups. This study was limited, however, by the smaller number of patients in each group^37^ (Table 3).

**Table 3:** Summary of articles examining the effects of probiotics in children with antibiotic-associated diarrhea.

Author (year) and study design	Setting	Population	Probiotic used	Dose	Duration	Outcome
Kotowska et al.^25^ (2005) RCT: probiotic versus placebo	3 children’s hospitals and 2 outpatient general pediatric clinics (not described) Warsaw, Nowa Deba, and Kielce, Poland	246 children (6 months–14 years) on antibiotics for otitis media or respiratory infections who are on antibiotics started within 24 h of enrollment	*Saccharomyces boulardii*	250 mg	Duration of antibiotic treatment	Lower rate of AAD in children receiving probiotics versus placebo. Rate of AAD in the probiotic group 4/119 (3.4%) versus 22/127 (17.3%) in the placebo group
Jindal et al.^26^ (2017) Randomized parallel study: probiotics and antibiotics versus antibiotics alone	Outpatient clinics at Muzaffarnagar Medical College (India)	600 children (6 months–12 years) prescribed antibiotics for common infections	*Saccharomyces boulardii*	2–3 billion CFU BID	7 days	Fewer patients in the probiotic groups developed diarrhea compared to antibiotics alone (16/300, 5.3% in the probiotics group, 72/300, 24% in antibiotics alone)
Shan et al.^27^ (2013) RCT: probiotic and antibiotic versus antibiotic alone	Inpatient hospital ward (Shengjing Hospital of China Medical University) Shenyang, China	283 patients (6 months–14 years) hospitalized and on antibiotics for acute lower respiratory tract infections	*Saccharomyces boulardii*	500 mg/day	2 weeks	Use of probiotic lowered risk of AAD. AAD in probiotics (6/139 (4.3%) versus placebo (28/144 (19.4%); RR: 0.22; 95% CI: 0.1–0.5)
Shan et al.^27^ (2013) RCT: second phase of the above study, probiotic and oral rehydration solution, or oral rehydration solution alone		42 patients enrolled in the above study in the antibiotics alone arm who developed diarrhea		500 mg/day	5 days	Lower stool frequency and shorter duration of AAD in the probiotic group. Mean duration of AAD in the probiotics group 2.31 ± 0.95 days versus 8.97 ± 1.07 days for the placebo group
Bin et al.^28^ (2015) RCT: antibiotics + probiotics versus antibiotics alone	Location of patient enrollment not specified	194 children between 22 months and 16 years treated with triple antibiotic therapy for *H. pylori* infection	*S. boulardii* CNCM I-745	500 mg/day	2 weeks	Decreased incidence of AAD in the probiotics versus control groups (11.8% versus 28.3%, *p* < 0.05)
Olek et al.^29^ (2017) RCT: probiotic versus placebo	Multiple outpatient primary healthcare centers (not described) in Poland	438 children (1–11 years) treated with antibiotic as an outpatient	*Lactobacillus plantarum* DSM9843	1 × 10^10^ CFU/day	Duration of antibiotics and an additional week after antibiotic discontinuation	No difference in the incidence of AAD. AAD occurred in 6/218 (2.8%) of children in the probiotic group and 9/220 (4.1%) of those in the placebo group
Georgieva et al.^30^ (2015) RCT: probiotic versus placebo	Inpatient ward at St Marina University Hospital, Varna, Bulgaria	97 children (3–12 years) inpatients on antibiotics for <48 h	*Lactobacillus reuteri* DSM 17938	1 × 10^8^ CFU /day	Duration of antibiotic plus additional 7 days	No difference in rate of diarrhea between groups (1/49 (2%) in the probiotics group, 1/48 (2%) in the placebo group, RR 0.98, 95% CI (0.93–1.02)
Kolodziej and Szajewska^31^ (2019) RCT: probiotics versus placebo	Two children’s hospitals: The Medical University of Warsaw and the General Hospital in Łukow (Poland)	247 children <18 years of age given oral or intravenous antibiotics	*Lactobacillus reuteri* DSM 17938	2 × 10^8^ CFU	Duration of antibiotics	Multiple outcomes included, but no difference occurred in any outcomes between the groups. Incidence of AAD 14/123 (11.4%) in the probiotic group, 8/124 (6.5%) in the placebo group, RR: 1.76, 95% CI (0.79–3.98)
Vanderhoof et al.^32^ (1999) RCT: probiotic versus placebo	Outpatient private primary care clinic (not further specified) Midwest, United States	188 children (6 months–10 years) on a 10-day course of antibiotics for infections of the respiratory tract, skin and soft tissue, or urinary tract	*Lactobacillus casei* sps. *rhamnosus* (*Lactobacillus GG*)	<12 kg: 10^9^ CFU/day >12 kg: 20^9^ CFU/day	Not stated	Decreased incidence of AAD in the probiotic (7/93, 8%) versus placebo groups (25/95, 26%). Reduced mean stool frequency, in the probiotic group (1.38, SEM 0.07) by day 10 of antibiotic compared to the placebo group (2.03, SEM 0.13)
Arvola et al.^33^ (1999) RCT: probiotics versus placebo	Clinics in the Health Care Centers in Tampere or Tampere University Hospital (Finland)	119 children (2 weeks–12.8 years) prescribed oral antibiotics for acute respiratory infections	*Lactobacillus GG* (ATCC 53103)	2 × 10^10^ CFU BID	Duration of antibiotic treatment	Lower rate of AAD in probiotic versus placebo: 3/61 (5%) patients in the probiotic group and 9/58 (16%) of patients in the placebo groups had diarrhea (*p* = 0.05). Reported treatment effect: −11% (−21 to 0%).
Ruszcynski et al.^34^ (2008) RCT: combination of probiotic strains versus placebo	2 children’s hospitals and one general pediatric outpatient clinic (not described) Warsaw, Poland	240 children (3 months–14 years) with common infections who started antibiotic treatment within 24 h of enrollment	*Lactobacillus rhamnosus* strains E/N, Oxy and Pen	2 × 10^9^ CFU BID	Duration of antibiotic treatment	Probiotic deceased risk of diarrhea but not specifically AAD (9/120 (7.5%) of those in the probiotic group and 20/120 (16.7%) in the placebo group developed diarrhea, RR 0.45 95% CI (0.2–0.9). 3/120 (2.5%) in the probiotic group and 9/120 (7.5%) in the placebo group develop AAD (RR 0.33 95% CI (0.1–1.06)
Szymański et al.^35^ (2008) RCT: combination of probiotics versus placebo	Multiple children’s hospitals and outpatient clinics (not described) Trzebnica, Kielce, and Cracow, Poland	78 children (5 months–16 years) with acute otitis media, respiratory tract infections, or urinary tract infection on antibiotics within 24 h	*Bifidobacterium* *longum* PL03	10^8^ (contained 1/3 of each strain) BID	Duration of antibiotic treatment	No difference in the incidence of AAD between the groups. AAD occurred in 1/40 (2.5%) in the probiotic group and 2/38 (5.3%) in the placebo group (RR 0.5 95% CI (0.06–3.5). Lower frequency of stool occurred in the probiotics groups (mean difference −0.3 (95% CI −0.5 to −0.07)
			*Lactobacillus rhamnosus* KL53A			
			*Lactobacillus* *plantarum* PL02			
Basnet et al.^36^ (2017) Antibiotic + probiotic versus antibiotic alone	Outpatient clinic at Manipal Teaching Hospital, Pokhara, Nepal	174 children (6 months–16 years) prescribed amoxicillin–clavulanate for respiratory infections	*Lactobacillus sporogenes*	5 × 10^8^ CFU BID	Not specified	Decreased incidence of AAD in the probiotic group (1/87 (1.1%)) versus antibiotic alone (11/87 (12.6%))
			*Streptococcus faecalis* T110	3 × 10^8^ CFU BID		
			*Clostridium butyricum* TO-A	2 × 10^7^ CFU BID		
			*Bacillus mesentericus* TO-A	1 × 10^7^ CFU BID		
Fox et al.^37^ (2015) RCT: probiotics in yogurt versus yogurt alone	General practices and pharmacies in Launceston (Tasmania, Australia)	70 children between 1 and 12 years prescribed oral broad-spectrum antibiotics as outpatients	Probiotic group: yogurt that contained: *Lactobacillus GG* (SDN: ATCC53103) *Bifidiobacterium lactis* (SDN: DSM15954) and *Lactobacillus acidophilus* (SDN: DSM13241) Placebo Group: Pasteurized yogurt that contained: *S. thermopolis* *Lactobacillus bulgaricus*	Probiotic group: *LGG*: 5.2 × 10^9^ CFU/day) *Bifidiobacterium:* 5.9 × 10^9^ CFU/day *Lactobacillus acidophilus*: 8.3 × 10^9^ CFU/day Placebo groups: *S. thermopolis*: 4.4 × 10^4^ CFU/day *Lactobacillus bulgaricus*: 1.2 × 10^3^ CFU/day	Duration of antibiotic treatment	Significant difference in rate of AAD: 1/34 children (3%) in the probiotic group and 27/36 children (75%) in the placebo group had any diarrhea. 0/34 (0%) in the probiotic group and 6/36 (17%) in the placebo group had severe diarrhea

*CFU* colony-forming unit, *BID* twice daily, *SDN* strain deposit number, *CI* confidence interval, *SEM* standard error of the mean.

We found several meta-analyses on this topic. A recent Cochrane Review, published in 2019, found an overall protective effect of probiotics and concluded that either *L. rhamnosus* or *S. boulardii* at a dose of 5–40 billion colony-forming units (CFUs)/day are the most appropriate choices for preventing AAD, with a number needed to treat to avoid one case of AAD of 9.^38^ Meta-analyses focused on the utility of *S. boulardii* or *LGG* supported these findings, with a number needed to treat of 10 for *S. boulardii* and 9 for *LGG*.^39,40^ These strains are also recommended by the European Society for Pediatric Gastroenterology, Hepatology, and Nutrition for use in the prevention of AAD.^41^ Similarly, a meta-analysis on *Bifidiobacterium* demonstrated efficacy in the prevention of AAD, although the authors did note that significant heterogeneity existed in the included studies.^42^

## Discussion

In our summary of the literature on the use of probiotics for common childhood indications including UTI prophylaxis, treatment of AD, and prevention of AAD, we found varying levels of evidence.

### Urinary tract infections

The dogma regarding the sterility of urine was recently challenged with the identification of the urinary microbiome.^43–45^ Since its initial description, a number of studies have shown decreased microbial diversity in the setting of an UTI, suggesting a potential role of the urinary microbiome in UTI pathogenesis.^46–48^ These studies that propose a role of the urinary microbiome in UTI pathogenesis are supported by older in vitro work. Studies suggest that *Lactobacillus* prevents the adherence of Gram-negative uropathogens to urothelial cells.^49,50^ Other work in this area demonstrated that oral administration of both *L. rhamnosus* GR-1 and *L. reuteri* (previously *fermentum*) RC-14 can colonize the vagina and decrease the presence of coliform bacteria,^51^ suggesting a potential role in decreasing risk of recurrent UTI. In addition, several animal models suggest that probiotic interventions may prevent UTIs.^52,53^

Most of the work in this area has focused on women, with little data focused on utility of probiotics in preventing UTI in children. In our analysis, we found only two RCTs regarding the use of probiotic interventions to prevent UTIs in children; however, neither intervention has been replicated in other settings. For children at risk for UTIs, preventive approaches would be beneficial, given the risk of renal scarring associated with febrile UTIs.

### Atopic dermatitis

The hypothesized role of the intestinal microbiome in AD occurs potentially through the interplay of the gastrointestinal (GI) microbiota and the immune system, which can affect the development of an imbalanced immune response. While the exact mechanism is not fully elucidated, one possibility is that the intestinal microbiota can either induce immune activation or trigger immune tolerance through cytokine stimulation.^54^ These changes, in turn, can exacerbate the T helper type 1 (Th1)/Th2 imbalance seen in AD, which is associated with increased secretion of IgE.^55^ Indeed, in vitro work demonstrated that a combination of non-specific strains of *L. acidophilus*, *L. casei*, *L. reuteri*, and *B. bifidum* (strains not specified) could exert anti-inflammatory properties through induction of T and B cell hyporesponsiveness.^56^

Further supporting the role of the intestinal microbiome in AD are several studies that have reported differences in stool microbiota between children with and without AD.^57–59^ Others did not find a difference in specific bacterial taxa but identified differences in functional genes related to immune development between children with and without AD using metagenomic analysis of stool.^60^ Finally, children with AD also have decreased levels of known anti-inflammatory metabolites, butyrate and propionate, in their stool.^57^

For children with AD, although there are RCTs of probiotic interventions that suggest some benefit, none of these studies have been replicated. Due to variation in the strains used, the population of interest, and the duration of treatment, comparisons are difficult. Further, several of the published works have limitations that make interpretation of their results difficult. Indeed, several studies suggest a benefit to the use of specific probiotic strains for treatment of children with AD; however, none of these studies have been reproduced. There is a need for large, confirmatory studies that replicate the findings of specific probiotic interventions for treatment of AD in children.

### Antibiotic-associated diarrhea

AAD is defined as the presence of unexplained diarrhea that occurs during a course of antibiotics. One mechanism through which intestinal dysbiosis leads to diarrhea is thought to be through altered digestion. Bacteria that are normally found within the colonic microbiome (i.e., *Clostridium*) ferment carbohydrates that were not absorbed in the small intestine into short-chain fatty acids once they pass into the colon. In the absence of these bacteria, which can occur following exposure to broad-spectrum antibiotics, the residual carbohydrates are not fermented and cause an osmotic diarrhea.^61,62^ Given the hypothesized relationship between antibiotics, dysbiosis, and ADD, there is much interest in the utility of probiotics to prevent or treat AAD.

We found studies that suggest that some specific probiotic strains may have some utility in preventing AAD, although in most children there are few significant consequences to AAD. Therefore, while probiotics may have some utility in preventing AAD, this may only have a limited clinical impact. Similarly, a 2019 Cochrane review on this topic found moderate evidence for the use of high-dose probiotics (≧5 billion CFUs/day) to prevent AAD, with the majority of the evidence supporting the use of either *L. rhamnosus GG* or *S. boulardii*.^38^

However, in the 2020 American Gastroenterological Association’s clinical practice guidelines on the use of probiotics, they do not comment on the use of probiotics for prevention of non-*C. difficile* colitis. The authors do, however, offer a conditional recommendation based on low-quality evidence to use probiotics to prevent *C. difficile* colitis in children on antibiotics.^63^

Much like the prior two conditions discussed, the significant heterogeneity in the literature on the use of probiotics in AAD is problematic when making recommendations regarding their use. There are also additional clinical considerations regarding the clinical use of probiotics for AAD. First, most patients may need to pay for probiotics “out of pocket,” which may present a financial burden. This concern is an important balancing measure to consider when deciding to initiate probiotics for the prevention of AAD, especially given that not every child given antibiotics will develop AAD. Overall, the number needed to treat to prevent AAD is approximately 11.^64,65^ While this is a reasonable number needed to treat, it is an important consideration when weighing the decision to prescribe probiotics for a patient for whom the cost of probiotics is prohibitive. Additionally, while probiotics are generally safe and well tolerated, there is the possibility that the increased complexity of the medication regimen may affect patient adherence with the antibiotic therapy. Indeed, the number of doses of medications has been demonstrated to be a factor in patient non-adherence with medication regimens in patients with chronic diseases.^66–68^ Finally, there is little oversight of the production and quality of commercially available probiotics. This was highlighted in a study of 14 commercially available probiotics, which found that only 1 of the 14 products studied contained the exact organisms listed on the label.^69^

## Society for Pediatric Research perspective

There are many areas of future research based on the current knowledge from clinical trials in these areas. These include assessing whether the native microbiota affects the clinical efficacy of a potentially therapeutic probiotic and exploring how the route of administration of probiotics affect changes in the microbiota in sites other than the GI tract. This issue is especially relevant for the urine microbiome and associated urological disease, as oral probiotics have an indirect path to the urine. Indeed, while some probiotics have been identified in the stool following oral administration,^70,71^ the same has not been seen in the urine. A recent trial of healthy pre-menopausal women who were given an oral combination of *L. rhamnosus GR-1* and *L. reuteri RC-14* for 40 days found that none of the strains within the oral probiotic were ever identified in the urine.^72^ This may partially explain results from a Cochrane review, where the authors concluded that oral probiotics did not prevent UTIs in people with neuropathic bladder,^73^ whereas a more recent paper found that intravesical administration of probiotics reduced urinary symptoms in people with neuropathic bladders.^74^ Further, *Lactobacillus* has been demonstrated to be effective against uropathogenic *Escherichia coli* in vitro, with mechanisms that require the presence of *Lactobacillus* within the urine.^75^ With many different potential probiotic interventions, a greater understanding of the effect of the route of administration and the interaction of specific strains with the microbiota may help select specific probiotic strains for clinical trials that might be more likely to be successful.

In our review, we found many small studies. There is a need to perform large, well-powered research studies to determine the utility of probiotics in these, and other, common pediatric conditions. Large pediatric studies have been conducted for some diseases, such as acute gastroenteritis.^76,77^ Larger samples sizes are critical to understand variation in responses to the probiotics and to identify whether there are subpopulations that may disproportionately benefit from their use. Larger sample sizes would also allow investigations into the interplay between the host microbiota and the probiotic strain and how this relates to clinical improvement. Standardization of variables, such as outcomes, use of concurrent therapies, and reporting of strain-level data, are needed to address the significant heterogeneity that is currently present in this body of research.

The need to report strain-level data of probiotics is critical given the increasing discussion in the literature regarding the concept of strain–disease specificity.^8,78–81^ In our review, we found that 19 of the 22 studies (86%) that used bacteria as probiotics included strain-specific information about the probiotic intervention. Strain-level data information will not only allow for direct comparisons between different studies but also to ensure that results are being accurately attributed to a specific strain, enabling translation to clinical practice.

The ability to identify components of the microbiome has led to new insights regarding the pathogenesis of disease. The correlation of dysbiosis and disease states has challenged the previous framework where a single pathogen is responsible for disease to a model focused on the effect of community dynamics. Changes in the microbiome exist in terms of community shifts. While these changes may be driven by a single organism, the result is manifest at the community level.^82^ For example, long-term exposure to *L. rhamnosus GG* may result in a shift in several components of the microbiome with increased abundance of *Prevotella*, *Lactococcus*, and *Ruminococcus* and decreased the proportion of *E. coli* in the intestinal microbiota.^83^ The administration of a specific strain by mouth leads to a shift in the intestinal microbiome, thus partially explaining why in vitro data are not always directly translated to the clinic.

Research into the potential therapeutic effects of probiotics remains an active area of investigation. Continued investigations into this area are critical to further our understanding of the pathophysiology of disease and ultimately enable our ability to improve child health.

## Disclaimer

This review represents the opinions and recommendations of the authors and do not represent consensus or clinical care guidelines.
